# Identification of Treatment Targets in Allergic Conjunctivitis Through Proteome‐Scale Mendelian Randomization Analysis

**DOI:** 10.1155/mi/6432686

**Published:** 2026-01-31

**Authors:** Hao Li, Yu Zhang, Yu Shang, Jinhui Dai, Shunmei Ji, Kunpeng Wu

**Affiliations:** ^1^ Department of Ophthalmology, Zhongshan Hospital, Fudan University, Shanghai, 200032, China, fudan.edu.cn; ^2^ Department of Ophthalmology, No.971 Hospital of the People’s Liberation Army Navy, Qingdao, 266071, China; ^3^ Department of Hematology, Huashan Hospital, Fudan University, Shanghai, 200040, China, fudan.edu.cn

**Keywords:** allergic conjunctivitis, circulating proteins, mendelian randomization, protein druggability, therapeutic targets

## Abstract

**Purpose:**

Allergic conjunctivitis (AC) is a condition with a rising prevalence that occasionally leads to irreversible visual impairment. However, few novel therapeutic targets have been identified for AC. The investigation aims to utilize the Mendelian randomization (MR) technique to explore the causal impacts exerted by the plasma proteome on AC.

**Methods:**

An evaluation was conducted on a two‐sample MR study utilizing 2,940 plasma proteins from the UK Biobank Pharma Proteomics Project (UKB‐PPP) to investigate their causal links with AC. Confirmation of these MR results was achieved using Summary‐data‐based MR (SMR) and Bayesian colocalization techniques. Moreover, evaluations concerning the druggability of proteins, interactions among proteins, and phenome‐wide MR (Phe‐MR) studies were conducted to ascertain the functionalities of the identified proteins implicated in causality.

**Results:**

MR analysis identified five circulating proteins (TLR1, ING1, RALY, CSF2, and ITGAM) whose genetically predicted plasma levels were significantly associated with AC risk (*P*_FDR_ < 0.05). Functional enrichment analysis of the identified proteins revealed statistically significant biologically relevant pathways, including macrophage activation and pathways of plasma membrane signaling receptor complex, suggesting underlying immune and receptor‐mediated mechanisms in AC. SMR and HEIDI validation supports four (TLR1, ING1, ITGAM, and CSF2) of five MR‐identified proteins as causal candidates for AC. Subsequent colocalization analysis provided further support for two credible proteins (ING1 and CSF2). Protein druggability suggests CSF2 and ING1 as novel and potentially druggable candidates for AC.

**Conclusions:**

Our research identified proteins that appear to have causal effects and could serve as potential therapeutic targets for AC.

## 1. Introduction

In the past several decades, there has been a notable increase in the prevalence of allergic conjunctivitis (AC), which is currently believed to affect between 10% and 20% of the global population [1]. AC is caused by an IgE‐mediated hypersensitivity reaction and characterized by conjunctival erythema, ocular pruritus, chemosis, and edema, which significantly impacts an individual’s quality of life [2]. What’s more, persistent exposure to allergens and ongoing ocular irritation frequently require repeated medical visits, resulting in significant economic consequences.

Theoretically, allergen avoidance and appropriate lifestyle factors may lead to the disappearance of symptoms through minimizing the contact of allergens with the eye. However, it is difficult to apply and insufficient to adequately control the symptoms. Hence, drugs, whether applied topically or administered systemically, are required to manage symptoms related to the eyes [3]. Currently, drugs used to treat AC mainly include H1‐receptor antagonists, decongestants, corticosteroids, nonsteroidal anti‐inflammatory drugs, calcineurin inhibitors, mast‐cell stabilizers, and allergen‐specific immunotherapy [4, 5]. However, none of the available agents is considered the ideal single drug, as each has its own specific drawbacks, such as ocular irritant effects, ocular pressure elevation, and development of cataracts. Therefore, it is critically important to prioritize research initiatives aimed at discovering and developing novel therapeutic agents for addressing AC.

Utilizing genetic variations as instrumental variables (IVs), Mendelian randomization (MR) analysis investigates the causal relationships between specific exposures and health outcomes. This methodological approach is increasingly applied to repurpose existing drugs and discover new therapeutic candidate targets [6, 7].

Plasma proteins are integral to numerous biological processes (BP) and therefore constitute attractive candidates for pharmacological intervention [8]. Advances in genome‐wide association studies (GWAS) have enabled large‐scale identification of single‐nucleotide polymorphisms (SNPs) that influence protein abundance in circulation. Such variants—known as protein quantitative trait loci (pQTLs)—provide a reliable genetic proxy for endogenous protein levels [9].

The integration of GWAS‐derived pQTL data with MR analysis provides an effective framework for prioritizing candidate drug targets. This integrated strategy enhances causal inference by reducing confounding and experimental bias, thus increasing the robustness and reliability of target nomination. It also improves efficiency by focusing resources on targets with strong genetic support, thereby streamlining the drug development pipeline [10].

Moreover, inclusion of phenome‐wide association studies (PheWAS) enables systematic investigation of the broad phenotypic consequences associated with genetically predicted modulation of protein targets. By applying PheWAS, both potential therapeutic benefits and unforeseen adverse phenotypes can be anticipated at an early stage, thereby informing target safety evaluation and guiding prioritization of candidate targets for further validation [11].

**Figure 1:**
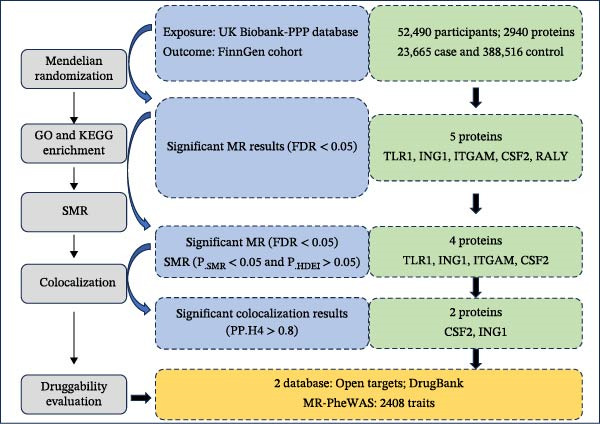
Flowchart of the study design.

In our study, plasma proteomic data from the UK Biobank Pharma Proteomics Project (UKB‐PPP) was utilized to conduct MR analysis with the aim of identifying circulating protein markers that may have a causal relationship with AC. To enhance the credibility of the MR findings, Bayesian co‐localization and SMR analysis were additionally performed. Moreover, the utilization of PheWAS and Protein–Protein Interaction (PPI) networks has facilitated the generation of valuable insights that could inform the development of therapeutic approaches directed at AC. The procedure of the study is illustrated in Figure 1. Consequently, plasma levels of inhibitor of growth protein 1 (ING1) and the granulocyte‐macrophage colony‐stimulating factor (CSF2) were newly identified and demonstrated to act as possible therapeutic targets for AC management.

## 2. Methods

### 2.1. Proteomic Data Source

Genetic variants of plasma proteins were leveraged, identified through extensive GWAS as part of the UKB‐PPP. This project utilized the Olink platform to analyze the proteomic profiles of a substantial cohort of 54,306 individuals from the UKB‐PPP, leading to the detection of 2,940 unique proteins. We utilized genetic variants of plasma proteins identified in comprehensive GWAS, specifically from the UKB‐PPP. The UKB‐PPP employed the Olink platform to perform proteomic analysis on a cohort comprising 54,306 participants from the UK Biobank, detecting a total of 2,940 distinct proteins (Supporting Information 1: Sheet 1) [12]. These pQTLs, discovered via GWAS associations, were subsequently used as genetic instruments for the assessment of each circulating protein.

### 2.2. The GWAS Data for AC

The foundational summary data for AC in the GWAS were sourced from the Finnish database accessible via https://r10.finngen.fi/. The dataset, labeled as finngen_R10_H7_ALLERGICCONJUNCTIVITIS, was specifically utilized for this analysis. The investigation encompassed a cohort of 23,665 cases alongside 388,516 controls, as detailed in Supporting Information 1: Sheet 1.

### 2.3. MR Analysis

In our study, MR was utilized alongside the two‐sample MR methodology to explore the association between AC and circulating plasma proteins. Initially, we selected IVs using criteria established in previous research: (1) SNPs with a significant association to the protein (*P* ≤ 5 × 10^−8^); (2) We applied a LD clumping cutoff of *r* ^2^ < 0.001; (3) SNPs and proteins not located within the Major Histocompatibility Complex (MHC) area of chromosome 6 (chr6 : 25.5–34.0 Mb); (4) an F‐statistic value of at least 10 (F = R^2^(n−2)/1−R^2^, where R^2^ = 2 × (1‐MAF) × MAF × β^2^) [13]. Initially, genetic markers for protein levels were matched with cis‐pQTLs derived from the genome‐wide association study (GWAS) focused on AC. Following this alignment, MR analysis was carried out.

The Inverse‐Variance Weighted (IVW) method was utilized as the primary approach for estimating the causal relationship between plasma proteins and AC, where multiple SNPs served as IVs [14]. In scenarios where only a single SNP served as the IV, calculations were based on the Wald ratio. Assessments of heterogeneity and the possibility of horizontal pleiotropy in the MR analyses were performed using the Cochran Q‐test and the MR‐Egger intercept. To enhance the credibility of the MR results, the directionality of the effects was cross‐validated through four alternative methods: the weighted median, weighted mode, simple mode, and the MR‐Egger. To explore potential reverse causality between exposure and outcome, Steiger analysis was employed.

To manage multiple comparisons effectively, the study employed a correction method based on the false discovery rate (FDR), designating a significance threshold at values less than 0.05. Depending on the level of heterogeneity observed, analysis models of IVW were selected accordingly: when *p* > 0.05 from Cochran’s Q‐test, indicating the absence of significant heterogeneity, the fixed‐effects IVW model was applied. When *p* < 0.05, reflecting substantial heterogeneity, the random‐effects IVW model was employed. This strategy ensures the robustness and accuracy of causal effect estimation. The MR analysis was performed utilizing the TwoSampleMR package within the R 4.4.0 environment.

### 2.4. Colocalization Study

In this study, a Bayesian colocalization approach was applied using R’s ‘coloc’ package to assess whether causal proteins and their linked variants within a specified genomic region were due to a shared causal variant, aiming to establish a genetic connection with the phenotype while ruling out LD or other confounders [15]. This investigation evaluated five distinct hypotheses: (1) H0 posits that the genetic variant has no association with any trait; (2) H1 suggests that a single causal variant is linked to a specific trait; (3) H2 indicates that a single causal variant influences a different trait; (4) H3 proposes that multiple causal variants within the region affect each trait independently; (5) H4 hypothesizes that one causal variant impacts both traits concurrently. The posterior probabilities for H3 (PPH3) and H4 (PPH4) were assessed in this context. Strong evidence for colocalization was concluded if PPH4 exceeded 0.8.

### 2.5. SMR Analysis

SMR analysis was utilized to investigate a potential causal linkage between specific proteins and AC [16]. The Heterogeneity in Dependent Instruments (HEIDI) test was applied to assess whether the correlations observed were attributable to shared genetic variants instead of genetic linkage. Both HEIDI and SMR analyses utilized the SMR software, setting the significance threshold at *p* < 0.05 for confirming a statistically significant causal effect. Conversely, a HEIDI test result with a *p*‐value greater than 0.05 indicated that the causality between the exposure and outcome might not be due to LD.

### 2.6. Pathway and Functional Enrichment Study

To explore the biological functions and the metabolic pathways associated with key proteins, extensive studies were performed using the Gene Ontology (GO) framework and the Kyoto Encyclopedia of Genes and Genomes (KEGG). The GO framework is categorized into three main domains: cellular components (CC), molecular functions (MF), and BP, each providing insights into different aspects of protein roles. This analysis evaluates the enrichment of each GO annotation by comparing the studied genes against the reference genome, thus generating enrichment analysis results. KEGG enrichment analysis, on the other hand, primarily focuses on identifying relevant metabolic pathways. Functional analysis was performed on all five causal proteins using both GO and KEGG pathways. The “clusterProfiler” and “tidyverse” R packages were employed for this analysis. Statistical significance was attributed to results displaying adjusted *p* *＜*0.05.

### 2.7. Evaluation of Druggability and Construction of PPI Networks

To assess the potential for therapeutic targeting of the identified causal proteins, a thorough investigation was conducted using the DrugBank resource (https://go.drugbank.com) to find drugs linked with these proteins. This method helps uncover opportunities for drug repurposing, where medications originally developed for other conditions might show efficacy in treating AC. To further investigate the pharmacological interventions for AC, the Open Targets platform (https://platform.opentargets.org) was utilized to identify currently deployed therapeutic agents. Additionally, the Search Tool for the Retrieval of Interacting Genes (STRING) database (https://string-db.org/) was employed to explore the PPI network. This analysis facilitates an enhanced understanding of the interconnections among proteins and how they align with the drugs targeting AC [17].

### 2.8. Phe‐MR Analysis

Phe‐MR studies were conducted to forecast potential adverse drug reactions and to predict the therapeutic effects of medications targeting specific proteins in the context of various diseases. This analysis utilized the Finngen_R10 database, examining two colocalization‐positive proteins (CSF2 and ING1) alongside 2408 traits. To ascertain causal connections between plasma proteins and specific traits, a FDR threshold of less than 0.05 was utilized.

## 3. Results

### 3.1. Proteome‐Wide MR Screening identifies 5 Proteins Causally Associated With AC

**Figure 2:**
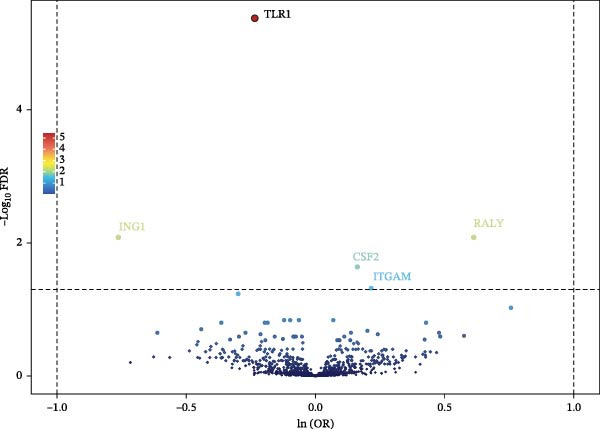
Results of MR on circulating proteins in relation to AC risk. The volcano plot of 5 plasma proteins versus risk of AC shows MR analyses of plasma proteins on AC risk using Wald ratios or IVW.

**Figure 3:**
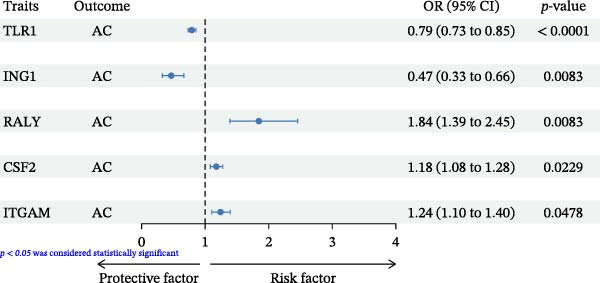
Forest plot results of circulating proteins in relation to AC. The error bars depict the AC OR for every 1 SD rise in protein expression, computed utilizing the Wald ratio (if 1 SNP) or the inverse variance weighted method (if > 1 SNP), and adjusted using FDR.

Following a rigorous application of screening criteria, 1925 distinct plasma proteins were ultimately selected for the subsequent MR analysis. IVW, or Wald ratio, was used as the main method. 5 proteins were proved to have significant results with AC (Figure 2 and Supporting Information 1: Sheet 2). Toll‐like receptor 1 (TLR1, OR = 0.791, 95% CI: 0.731–0.855,  _*p* *F* *D* *R*_ = 4.22E − 06) and ING1 (OR = 0.466, 95% CI: 0.327–0.665, _*p*FDR_ = 8.26E‐03) were the protective factor of AC. While RNA‐binding protein Raly (RALY, OR = 1.845, 95% CI: 1.388–2.454, _*P*FDR_ = 8.26E‐03), CSF2 (OR = 1.176, 95% CI: 1.083–1.277, _*p*FDR_ = 0.023), and integrin alpha‐M (ITGAM, OR = 1.241, 95% CI: 1.104–1.395, _*p*FDR_ = 0.048) were the risk factors of AC (Figure 3). The MR‐Egger intercept test showed no signs of horizontal pleiotropy, as indicated by a *p*‐value greater than 0.05. Similarly, the Cochran Q‐test revealed no significant heterogeneity (*p* > 0.05), as detailed in Supporting Information 1: Sheet 3. Consistency in the effect direction across four distinct MR methodologies ‐ MR‐Egger, Weighted median, Weighted mode, and IVW/Wald ratio—underscores the robustness of our findings.

### 3.2. Enrichment Analysis of AC‐Associated Proteins

**Figure 4:**
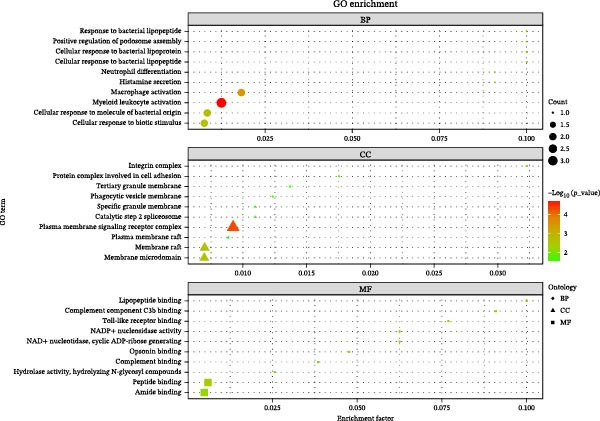
GO enrichment analysis of five identified plasma proteins for therapy of AC. The top 10 enriched GO terms are listed.

**Figure 5:**
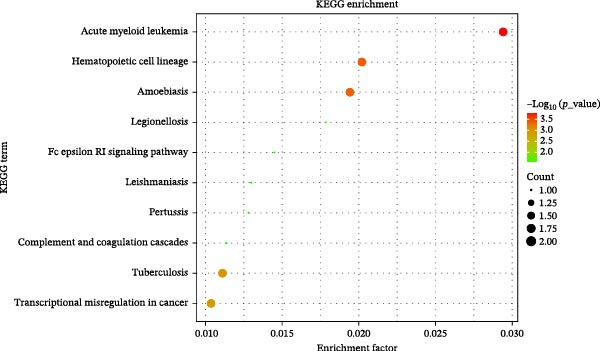
KEGG enrichment analysis of five identified plasma proteins for therapy of AC. The top 10 enriched KEGG terms are listed.

To elucidate the biological functions of the proteins identified (TLR1, ING1, RALY, CSF2, and ITGAM), analyses of GO and KEGG pathways were performed to enrich the understanding. The GO annotation results for the top 10 pathways indicate enrichment in BP such as macrophage activation, myeloid leukocyte activation, cellular responses to molecules of bacterial origin, and cellular responses to biotic stimuli. CC were predominantly associated with plasma membrane signaling receptor complexes, membrane rafts, and membrane microdomains. MF analysis revealed significant involvement of these proteins in peptide and amide binding activities (Figure 4). Analysis via the KEGG pathway database shows that these proteins are predominantly associated with pathways linked to acute myeloid leukemia, hematopoietic cell lineage, amebiasis, tuberculosis, and transcriptional dysregulation in cancers (Figure 5).

### 3.3. SMR and HEIDI Analysis Further Validate Four of Five MR‐identified Proteins as Causal Candidates for AC

To further substantiate the causal association between the five identified proteins and AC, a series of SMR analyses coupled with HEIDI tests were conducted. The results revealed that four (TLR1, ING1, ITGAM, CSF2) out of the five proteins successfully met the criteria in both the SMR analysis and HEIDI test (with *p*SMR < 0.05 and _*p*HEIDI_ > 0.05, Supporting Information 1: Sheet 4). Although RALY showed a nominally significant association in the SMR analysis, it failed to pass the HEIDI test (_*p*HEIDI_ = 0.002889), indicating heterogeneity among instrumental variants.

### 3.4. Bayesian Colocalization Analysis

An in‐depth colocalization analysis was performed using the coloc package in R to examine whether the genetic signals for AC and the corresponding pQTLs arose from shared causal variants. For CSF2, the posterior probability supporting a shared causal variant (PPH4 = 0.813) was substantially higher than the probability indicating distinct causal variants (PPH3 = 0.097). Similarly, for ING1, the probability of a shared causal variant (PPH4 = 0.851) clearly exceeded that of separate causal variants (PPH3 = 0.082). This marked contrast between PPH4 and PPH3 provides strong evidence that CSF2 and ING1 share the same causal genetic variants with AC rather than reflecting linkage (Supporting Information 1: Sheet 5 and Supporting Information 2: Figure S1).

### 3.5. Phe‐MR Study of Causal Proteins Upon 2408 Disease Traits

A Phe‐MR study was conducted on causal proteins across 2408 disease traits in the FinnGen study database to explore the potential therapeutic effects of drugs targeting these proteins on other diseases and to predict any potential adverse drug reactions. Supporting Information 1: Sheet 6 showed the result of the Phe‐MR analysis. After FDR correction, CSF2 was found to be related to asthma, coronary revascularization, weight, varicose veins, asthma‐related pneumonia, coronary angioplasty, chronic lower respiratory diseases, wet age‐related macular degeneration, asthma‐related infections, and angina pectoris. While ING1 corresponds to no specific disease or trait by IVW.

### 3.6. Druggability of Identified Proteins

We searched the drug database (https://platform.opentargets.org/) to find the targets of current medications. 19 proteins were related to current AC medications (Supporting Information 1: Sheet 7). And there was no evidence showing that the 4 proteins identified were the direct targets for AC. PPI network research showed close interactions between 3 causal proteins (ITGAM, CSF2, TLR1) and target proteins currently employed for AC drug development, indicating circulating proteins identified by MR were closely related to drug targets (Supporting Information 2: Figure S2). In our study, CSF2 and ING1 were newly discovered possible drug targets for AC. No information was available for 5 causal proteins in the drug database.

## 4. Discussion

In this study, MR was employed to assess correlations between 1925 plasma proteins and the risk of AC development. MR analysis identified that diminished levels of TLR1 and ING1, predicted by genetic markers, along with increased levels of RALY, CSF2, and ITGAM, are associated with an increased risk of developing AC. However, subsequent validation using SMR analysis coupled with the HEIDI test demonstrated that only four proteins (TLR1, ITGAM, ING1, and CSF2) showed robust evidence consistent with a shared causal variant underlying both protein levels and AC risk. Although RALY exhibited a nominally significant association in the initial MR analysis, it failed to pass the HEIDI test, indicating heterogeneity among instrumental variants and suggesting that the observed association is unlikely to be driven by a single shared causal variant. Furthermore, CSF2 and ING1 have the causative variants linked to AC using colocalization analysis. Collectively, these results offer direction and fresh insights for targeted therapeutics in AC therapy and prevention.

CSF2, also known as granulocyte‐macrophage colony‐stimulating factor (GM‐CSF), is implicated in macrophage polarization [18] and eosinophils activation [19], which are vital processes in the development of AC. The polarization of macrophages towards the M2 phenotype exacerbates allergic inflammation in murine models of experimental AC induced by short ragweed pollen [20]. Although the present study does not directly address the impact of CSF2 on macrophage function within the context of AC, research by Anthony et al. has demonstrated the pathological significance of CSF2 in enhancing pro‐inflammatory functions of monocytes in antineutrophil cytoplasmic antibody‐induced glomerulonephritis [21]; this evidence implies a potential regulatory role for CSF2 in macrophage activation during AC progression. Moreover, GM‐CSF induces eosinophil production of IL‐4 and chemokines in experimental AC [22], indicating the pathogenic role of CSF2 in AC.

Unlike conventional treatments for allergic conditions such as antihistamines or mast‐cell stabilizers, which suppress downstream effector events such as histamine release or mast‐cell degranulation, CSF2 targets immune regulation at a more upstream level [23]. Because of this upstream immunomodulatory role, therapeutic strategies directed at CSF2 may modulate the initiation and amplification of allergic inflammation rather than merely alleviating downstream symptoms. As a result, CSF2‐targeted intervention could potentially yield broader and more durable control of immune responses underlying AC, compared with agents that act solely on histamine signaling or mast‐cell degranulation. Research outcomes have demonstrated a significant correlation between elevated CSF2 levels, as determined genetically, and an increased likelihood of AC development, which aligns with observations from experimental research. This evidence, coupled with our study’s results, provides strong support for the hypothesis that CSF2 may play a role in influencing AC risk, making it a valuable direction for further investigation.

ING proteins, evolutionarily conserved components of HAT and HDAC complexes, modify chromatin architecture, subsequently regulating gene expression [24]; these modifications have been functionally associated with cell cycle arrest and apoptosis [25]. Dysregulated expression of the ING1 gene has been identified as a pivotal factor in the onset of multiple cancer types. Although ING1 is classically recognized as a tumor suppressor and chromatin remodeling factor [26, 27], its epigenetic regulatory capacity could conceivably influence immune regulation and inflammatory responses [28, 29]. Epigenetic remodeling controls chromatin architecture in innate immune cells, shaping cell‐type‐specific gene expression, including immune‐ and inflammation‐related genes [28, 29]. Given the growing evidence linking dysregulated chromatin remodeling to chronic inflammation and immune disorders [28], ING1 emerges as a plausible regulator of immune homeostasis relevant to AC. However, its regulatory role in allergic diseases has not been adequately investigated. Our studies reveal a significant link between ING1 and the exacerbation of allergic disorders, confirmed through colocalization studies. Given the study that identified a causal relationship between ING1 and AC risk, increasing circulating ING1 may be a viable strategy for lowering AC risk.

ITGAM, also known as CD11b, was recognized as a risk factor for AC. The ’predisposing’ variant of ITGAM (rs1143679, Arg77His) is predicted to alter the tertiary structure of its ligand‐binding domain, consequently increasing the risk of developing autoimmune diseases like systemic lupus erythematosus (SLE) [30] and systemic sclerosis [31]. Whether variation caused by ITGAM loss of protein binding function would lead to altered circulating ITGAM is still undetermined. Additional investigations are required to dissect the concentration and structure variation of ITGAM in AC etiology. Given the study’s identification of a causal relationship between ITGAM and AC risk, circulating ITGAM reduction strategies may be a viable way to lower AC risk.

Our proteome‐wide MR analysis also identified TLR1 as a protein whose genetically predicted lower plasma level was associated with increased risk of AC. TLR1/2 heterodimer recognizes bacterial lipoproteins, triggering downstream signaling cascades that activate innate immune responses and the release of inflammatory mediators [32]. To date, most studies on TLR involvement in AC and ocular allergy have focused on other TLR family members (e.g., TLR4) rather than TLR1. For example, activation of TLR4 has been shown to modulate the development of experimental AC, and LPS‐mediated TLR4 signaling can alter the balance between Th1/Th2 immune responses in ocular allergy models [33]. There remains a lack of direct experimental evidence detailing how TLR1 influences conjunctival immune responses or allergic inflammation in AC. TLR1 represents a candidate derived from our genetic screening with a biological basis for involvement in AC, but its role remains speculative until confirmed by dedicated immunological and functional experiments.

This study is subject to several limitations. While MR analysis offers valuable insights, core limitations inherent to the MR framework must be acknowledged. Violations such as horizontal pleiotropy or linkage disequilibrium can bias causal estimates, even when sensitivity analyses are applied. The dataset analyzed consists solely of GWAS information from individuals of European ancestry, thus limiting the applicability of our conclusions across diverse ethnic groups. Moreover, the scope of statistical power remains constrained by the modest dimensions of the largest available GWAS dataset for AC, heightening the susceptibility to bias. Comprehensive fundamental and clinical research is imperative to elucidate the molecular mechanisms underlying the effects of the investigated protein.

Our study is based on summary‐level GWAS data and cannot determine whether the predicted alterations in protein levels actually occur in patients with AC, nor can it clarify their tissue‐specific expression or functional role in conjunctival or immune cells. Therefore, our findings should be considered as hypothesis‐generating candidate associations rather than definitive proof of causation or therapeutic suitability. To translate these findings into biologically and clinically relevant targets, future studies are required. We recommend measuring CSF2 and ING1 protein levels in ocular surface–related samples (tear fluid, conjunctival tissue, blood) from well‐characterized AC patients and matched controls. In vitro experiments using ocular surface epithelial or immune cells should examine whether modulation of these proteins affects inflammatory mediator production, immune cell activation, or recruitment. Finally, in vivo models of ocular allergy could test whether perturbation of CSF2 or ING1 alters disease onset or severity.

In conclusion, we have effectively expanded upon the existing biomarkers of AC and acquired a better comprehension of its etiology. We found five plasma proteins linked to AC using MR analysis, with CSF2 and ING1 strongly colocalized with AC. Protein druggability and PPI networks newly indicated that CSF2 and ING1 may represent future intervention and treatment targets for AC.

## Author Contributions

Hao Li and Yu Zhang data collection, writing, and analysis. Kunpeng Wu and Jinhui Dai conceptualization, supervision, and project administration. Shunmei Ji and Yu Shang revised the manuscript.

## Funding

This work was supported by the National Natural Science Foundation of China (Grants 82100232 and 82401297).

## Disclosure

All authors have read and agreed to the published version of the manuscript.

## Ethics Statement

All GWAS statistics used in this study were from published work. Ethics committees of all original institutions approved all of the GWASs following the tenets of the Declaration of Helsinki.

## Conflicts of Interest

The authors declare no conflicts of interest.

## Supporting Information

Additional supporting information can be found online in the Supporting Information section.

## Supporting information


**Supporting Information 1** Supporting Information Sheet 1: Summary statistics of allergic conjunctivitis GWAS from FinnGen (23,665 cases and 388,516 controls). Supporting Information Sheet 2: MR analysis results: plasma proteins significantly associated with allergic conjunctivitis. Supporting Information Sheet 3: Results of MR‐Egger intercept and Cochran Q tests indicating no horizontal pleiotropy or heterogeneity. Supporting Information Sheet 4: SMR and HEIDI test results supporting causal associations between four plasma proteins and allergic conjunctivitis. Supporting Information Sheet 5: Bayesian colocalization analysis for pQTL and AC. Supporting Information Sheet 6: Phe‐MR analysis results of causal proteins across 2408 disease traits in the Finngen study. Supporting Information Sheet 7: Druggability assessment of identified proteins based on current AC medications.

**Supporting Information 2** Supporting information Figure S1: Bayesian colocalization analysis for pQTL and AC. The x‐axis represents the −log10 P GWAS of pQTL, and the y‐axis shows −log10 P GWAS of corresponding GWAS AC. Supporting Information Figure S2: Protein–Protein interaction network among the causal proteins and current AC medication targets. 3 of 5 identified proteins are shown in the figure while two proteins, ING1 and RALY, have no interaction with all other proteins and are deleted in this analysis.

## Data Availability

The GWAS data used to support the findings of this study are available from the corresponding author upon request.
