# Supplementary material for: Identification of Treatment Targets in Allergic Conjunctivitis Through Proteome‐Scale Mendelian Randomization Analysis
Source: Mediators Inflamm. 2026 Jan 31;2026:6432686. doi: 10.1155/mi/6432686 (PMC12860419; doi:10.1155/mi/6432686)
Supplement: Supplementary file 1 — Supporting Information 1 Supporting Information Sheet 1: Summary statistics of allergic conjunctivitis GWAS from FinnGen (23,665 cases and 388,516 controls). Supporting Information Sheet 2: MR analysis results: plasma proteins significantly associated with allergic conjunctivitis. Supporting Information Sheet 3: Results of MR‐Egger intercept and Cochran Q tests indicating no horizontal pleiotropy or heterogeneity. Supporting Information Sheet 4: SMR and HEIDI test results supporting causal associations between four plasma proteins and allergic conjunctivitis. Supporting Information Sheet 5: Bayesian colocalization analysis for pQTL and AC. Supporting Information Sheet 6: Phe‐MR analysis results of causal proteins across 2408 disease traits in the Finngen study. Supporting Information Sheet 7: Druggability assessment of identified proteins based on current AC medications. [file MI-2026-6432686-s002.docx]

**
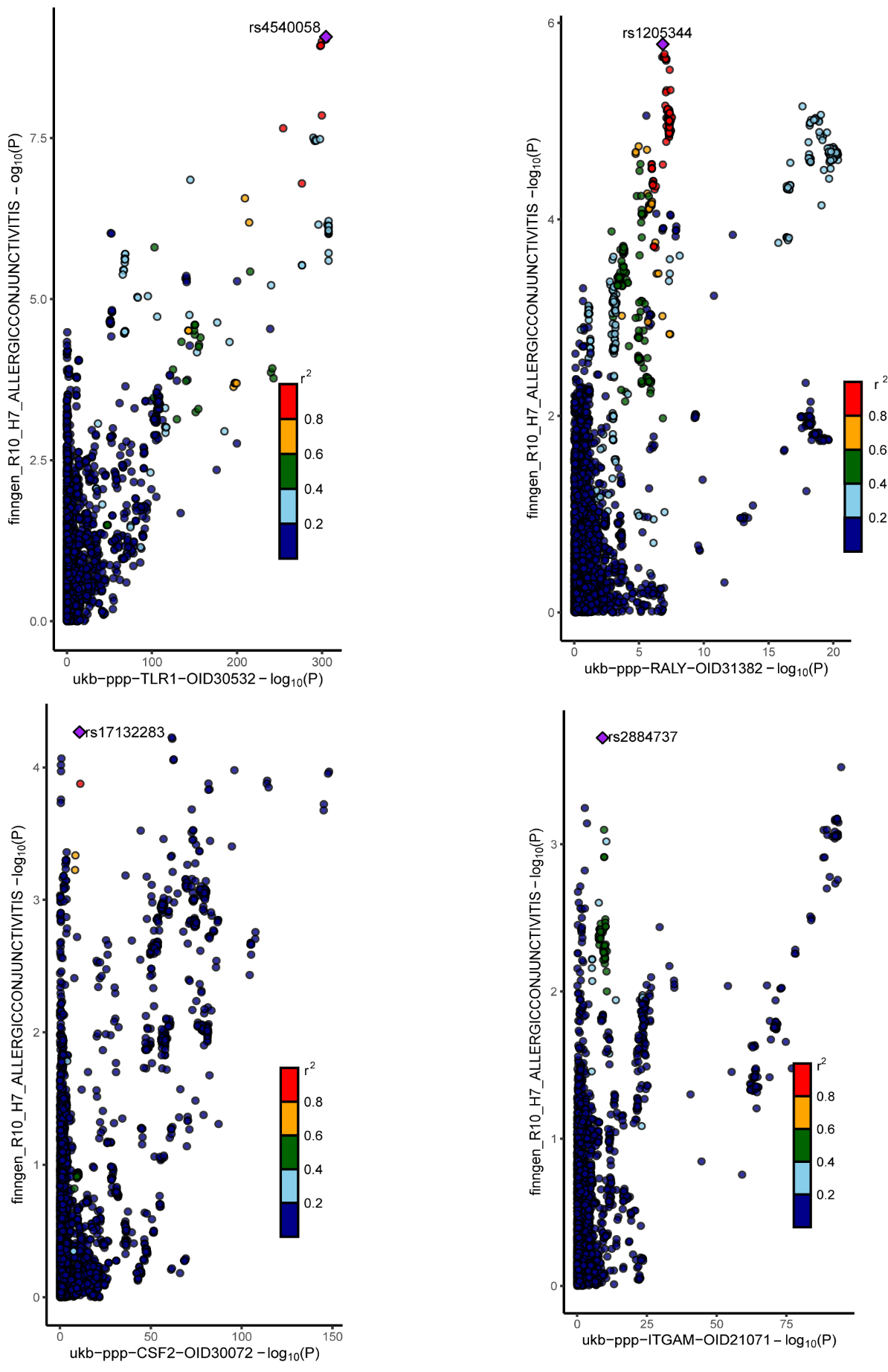
**

**
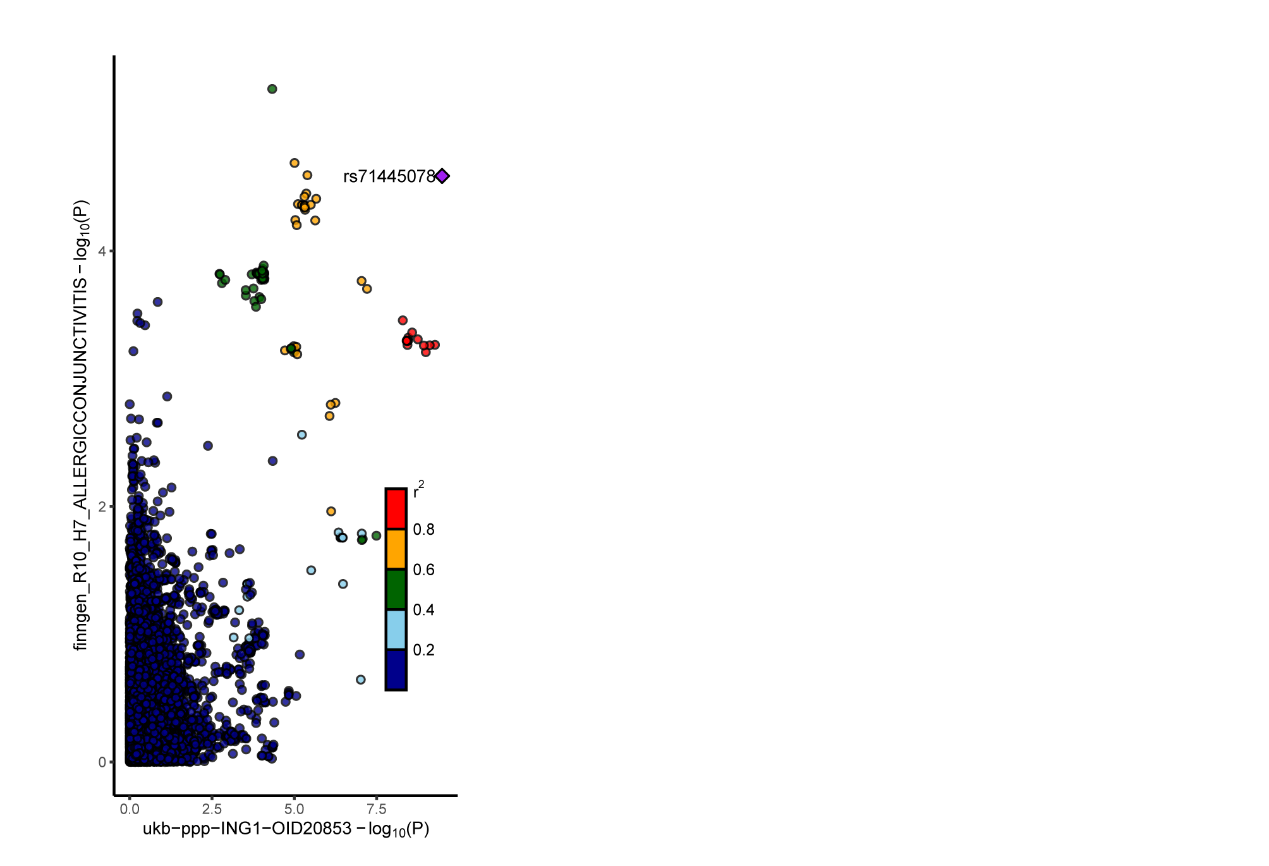
**

**Supplementary Fig.1** Bayesian colocalization analysis for pQTL and AC. The x-axis represents the −log10 P GWAS of pQTL, and the y-axis shows −log10 P GWAS of corresponding GWAS AC.


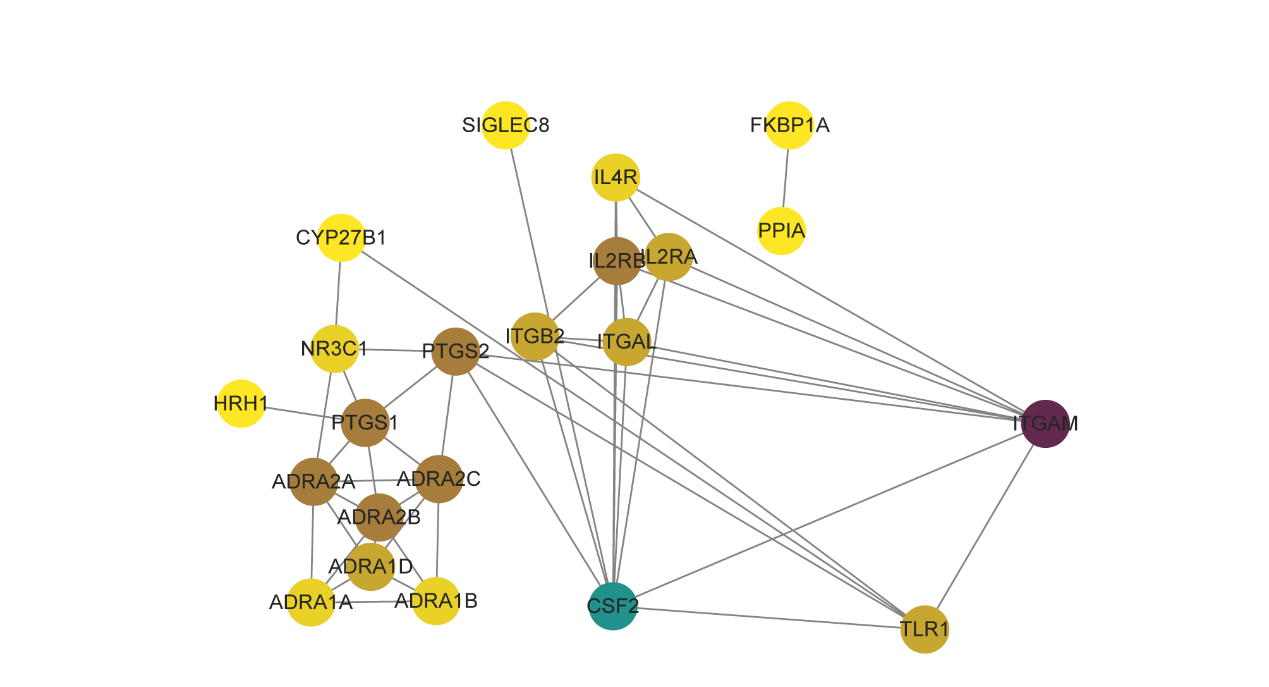


**Supplementary Fig.2** Protein-Protein interaction network among the causal proteins and current AC medications targets. 3 of 5 identified proteins are showed in figure while two proteins, ING1 and RALY, have no interaction with all other proteins and are deleted in this analysis.
