# Supplementary material for: Identification of Treatment Targets in Allergic Conjunctivitis Through Proteome‐Scale Mendelian Randomization Analysis
Source: Mediators Inflamm. 2026 Jan 31;2026:6432686. doi: 10.1155/mi/6432686 (PMC12860419; doi:10.1155/mi/6432686)
Supplement: Supplementary file 2 — Supporting Information 2 Supporting information Figure S1: Bayesian colocalization analysis for pQTL and AC. The x‐axis represents the −log10 P GWAS of pQTL, and the y‐axis shows −log10 P GWAS of corresponding GWAS AC. Supporting Information Figure S2: Protein–Protein interaction network among the causal proteins and current AC medication targets. 3 of 5 identified proteins are shown in the figure while two proteins, ING1 and RALY, have no interaction with all other proteins and are deleted in this analysis. [file MI-2026-6432686-s001.pdf]

Exposure/Outcome	Consortium/First author	Participants	Pubmed ID/Web source
circulating proteins levels	Benjamin B Sun	54219	37794186
Allergic conjunctivitis	The FinnGen study	412181	https://r10.finngen.fi/

exposure	outcome	method	nsnp	beta	se	pval	or	or_lci95	or_uci95	p_fdr	egger intercept pval	Hetero geneity Q pval
**FinnGen Cohort**												
TLR1	Allergic conjunctivitis	Inverse variance weighted	8	-0.23481	0.039685	0.00000000328	0.790723	0.731551	0.854682	0.00000422	0.89274751	0.1705874
ING1	Allergic conjunctivitis	Wald ratio	1	-0.76307	0.181372	0.0000259	0.466234	0.326751	0.665259	0.00826065	NA	NA
RALY	Allergic conjunctivitis	Wald ratio	1	0.612725	0.145324	0.0000248	1.845453	1.388037	2.453606	0.00826065	NA	NA
ITGAM	Allergic conjunctivitis	Wald ratio	1	0.215781	0.059679	0.000299525	1.240831	1.103858	1.394801	0.04784908	NA	NA
CSF2	Allergic conjunctivitis	Wald ratio	1	0.162272	0.041904	0.000107754	1.17618	1.083438	1.276861	0.02295166	NA	NA

exposure	SNP	effect_allele.exposure	other_allele.exposure	beta.exposure	eaf.exposure	originalname.outcome	se.exposure	pval.exposure	F
TLR1	rs137879422	C	T	-0.138919	0.0272815	Allergic conjunctivitis	0.0245586	1.54358E-08	33.81359
TLR1	rs150023553	A	G	0.162771	0.0204158	Allergic conjunctivitis	0.0283487	9.37109E-09	34.98567
TLR1	rs183976409	A	G	-0.26147	0.0136383	Allergic conjunctivitis	0.034257	2.30091E-14	60.77252
TLR1	rs4833108	C	G	0.257912	0.0264057	Allergic conjunctivitis	0.0249612	5.02227E-25	113.1812
TLR1	rs73234989	A	G	0.450595	0.156671	Allergic conjunctivitis	0.0107627	1E-200	1869.714
TLR1	rs75293998	T	G	0.159497	0.026836	Allergic conjunctivitis	0.0241181	3.7627E-11	43.8787
TLR1	rs79981910	T	A	0.178874	0.0238523	Allergic conjunctivitis	0.0263885	1.21451E-11	49.21022
TLR1	rs9990980	A	C	0.481834	0.0132357	Allergic conjunctivitis	0.0342432	5.7306E-45	201.2169
ING1	rs71445078	G	T	-0.0625263	0.185255	Allergic conjunctivitis	0.00994611	3.24691E-10	38.82525
RALY	rs1007090	T	C	0.0749947	0.648396	Allergic conjunctivitis	0.00795905	4.40251E-21	86.83519
ITGAM	rs11150613	T	C	0.163623	0.676047	Allergic conjunctivitis	0.00789882	2.54273E-95	399.7027
CSF2	rs25882	C	T	0.243807	0.206606	Allergic conjunctivitis	0.00938271	7.379E-149	653.0833

Supplementary Sheet4

Gene	P_SMR	P_HEIDI
TLR1	5.87834E-17	0.1458
ING1	4.72E-04	0.0604
RALY*	0.000118817	0.002889
ITGAM	0.000368238	0.213132
CSF2	1.28E-04	0.420546

* SMR significant, HEIDI failed

Supplementary Sheet5

Proteins	PP.H3	PP.H4
TLR1	0.91259	0.087197
ING1	0.081694	0.850704
RALY	0.342799	0.641869
ITGAM	0.072068	0.661163
CSF2	0.096533	0.812729

id.exposure	id.outcome	outcome	exposure	method	nsnp	b	se	pval	or	or_lci95	or_uci95	p_fdr
ukb-ppp-CSF2-OID30072	finngen_R10_J10_ASTHMACOPDKELA	Asthma/COPD (KELA code 203) || id:finngen_R10_J10_ASTHMACOPDKELA	CSF2_P04141_OID30072_v1_Cardiometabolic_II || id:ukb-ppp-CSF2-OID30072	Inverse variance weighted	9	0.174679	0.033758	2.29E-07	1.190864	1.11462	1.272323	0.00055
ukb-ppp-CSF2-OID30072	finngen_R10_I9_REVASC	Coronary revascularization (ANGIO or CABG) || id:finngen_R10_I9_REVASC	CSF2_P04141_OID30072_v1_Cardiometabolic_II || id:ukb-ppp-CSF2-OID30072	Inverse variance weighted	9	-0.18996	0.038816	9.88E-07	0.826988	0.766405	0.89236	0.00119
ukb-ppp-CSF2-OID30072	finngen_R10_WEIGHT_IRN	Weight, inverse-rank normalized || id:finngen_R10_WEIGHT_IRN	CSF2_P04141_OID30072_v1_Cardiometabolic_II || id:ukb-ppp-CSF2-OID30072	Inverse variance weighted	9	-0.04437	0.010003	9.20E-06	0.956603	0.938029	0.975544	0.007384
ukb-ppp-CSF2-OID30072	finngen_R10_I9_VARICVE	Varicose veins || id:finngen_R10_I9_VARICVE	CSF2_P04141_OID30072_v1_Cardiometabolic_II || id:ukb-ppp-CSF2-OID30072	Inverse variance weighted	9	0.150251	0.035244	2.02E-05	1.162125	1.084558	1.245241	0.012134
ukb-ppp-CSF2-OID30072	finngen_R10_ASTHMA_PNEUMONIA	Asthma-related pneumonia || id:finngen_R10_ASTHMA_PNEUMONIA	CSF2_P04141_OID30072_v1_Cardiometabolic_II || id:ukb-ppp-CSF2-OID30072	Inverse variance weighted	9	0.183636	0.045892	6.30E-05	1.201578	1.098216	1.314668	0.024241
ukb-ppp-CSF2-OID30072	finngen_R10_I9_ANGIO	Coronary angioplasty || id:finngen_R10_I9_ANGIO	CSF2_P04141_OID30072_v1_Cardiometabolic_II || id:ukb-ppp-CSF2-OID30072	Inverse variance weighted	9	-0.18522	0.046594	7.03E-05	0.830922	0.758401	0.910378	0.024241
ukb-ppp-CSF2-OID30072	finngen_R10_I9_DISVEINLYMPH	Diseases of veins, lymphatic vessels and lymph nodes, not elsewhere classified || id:finngen_R10_I9_DISVEINLYMPH	CSF2_P04141_OID30072_v1_Cardiometabolic_II || id:ukb-ppp-CSF2-OID30072	Inverse variance weighted	9	0.106757	0.026993	7.65E-05	1.112664	1.055327	1.173116	0.024241
ukb-ppp-CSF2-OID30072	finngen_R10_J10_LOWCHRON	Chronic lower respiratory diseases || id:finngen_R10_J10_LOWCHRON	CSF2_P04141_OID30072_v1_Cardiometabolic_II || id:ukb-ppp-CSF2-OID30072	Inverse variance weighted (multiplicative random effects)	9	0.153947	0.039045	8.05E-05	1.166429	1.080494	1.259198	0.024241
ukb-ppp-CSF2-OID30072	finngen_R10_WET_AMD	Wet age-related macular degeneration || id:finngen_R10_WET_AMD	CSF2_P04141_OID30072_v1_Cardiometabolic_II || id:ukb-ppp-CSF2-OID30072	Inverse variance weighted	9	0.346901	0.089001	9.71E-05	1.414677	1.188224	1.684288	0.02598
ukb-ppp-CSF2-OID30072	finngen_R10_ASTHMA_INFECTIONS	Asthma-related infections || id:finngen_R10_ASTHMA_INFECTIONS	CSF2_P04141_OID30072_v1_Cardiometabolic_II || id:ukb-ppp-CSF2-OID30072	Inverse variance weighted (multiplicative random effects)	9	0.206145	0.053961	0.000133	1.228931	1.105592	1.36603	0.032104
ukb-ppp-CSF2-OID30072	finngen_R10_I9_ANGINA	Angina pectoris || id:finngen_R10_I9_ANGINA	CSF2_P04141_OID30072_v1_Cardiometabolic_II || id:ukb-ppp-CSF2-OID30072	Inverse variance weighted	9	-0.11801	0.031925	0.000219	0.888691	0.834787	0.946077	0.047891

Supplementary Sheet7

disease Id	diseaseId	diseaseName	drugId	drugName	type	mechanismOfAction	actionType	symbol	name	phase	status	source
EFO_0001365	EFO_0007141	allergic conjunctivitis	CHEMBL1716	CYPROHEPTADINE HYDROCHLORIDE	Small molecule	Histamine H1 receptor antagonist	Antagonist	HRH1	histamine receptor H1	4		[illegible]
EFO_0001365	EFO_0007141	allergic conjunctivitis	CHEMBL1201014	PREDNISOLONE SODIUM PHOSPHATE	Small molecule	Glucocorticoid receptor agonist		NR3C1	nuclear receptor subfamily 3 group C member	4	Completed	[illegible]
EFO_0004683	EFO_0007141	allergic conjunctivitis	CHEMBL1201758	BEPOTASTINE	Small molecule	Histamine H1 receptor antagonist	Antagonist	HRH1	histamine receptor H1	4		[illegible]
EFO_0004683	EFO_0007141	allergic conjunctivitis	CHEMBL1200491	EPINASTINE HYDROCHLORIDE	Small molecule	Histamine H1 receptor antagonist	Antagonist	HRH1	histamine receptor H1	4		[illegible]
EFO_0001365	EFO_0007141	allergic conjunctivitis	CHEMBL1189432	OLOPATADINE	Small molecule	Histamine H1 receptor antagonist	Antagonist	HRH1	histamine receptor H1	4	Completed	[illegible]
EFO_0004683	EFO_0007141	allergic conjunctivitis	CHEMBL1200865	LOTEPREDNOL ETABONATE	Small molecule	Glucocorticoid receptor agonist		NR3C1	nuclear receptor subfamily 3 group C member	4		[illegible]
EFO_0001365	EFO_0007141	allergic conjunctivitis	CHEMBL384467	DEXAMETHASONE	Small molecule	Glucocorticoid receptor agonist		NR3C1	nuclear receptor subfamily 3 group C member	4		[illegible]
EFO_0004683	EFO_0007141	allergic conjunctivitis	CHEMBL1200865	LOTEPREDNOL ETABONATE	Small molecule	Glucocorticoid receptor agonist		NR3C1	nuclear receptor subfamily 3 group C member	4	Completed	[illegible]
EFO_0001365	EFO_0007141	allergic conjunctivitis	CHEMBL1706	NAPHAZOLINE HYDROCHLORIDE	Small molecule	Adrenergic receptor alpha agonist		ADRA1D	adrenoceptor alpha 1D	4	Unknown status	[illegible]
EFO_0001365	EFO_0007141	allergic conjunctivitis	CHEMBL639	AZELASTINE	Small molecule	Histamine H1 receptor antagonist	Antagonist	HRH1	histamine receptor H1	4		[illegible]
EFO_0001365	EFO_0007141	allergic conjunctivitis	CHEMBL1719	OLOPATADINE HYDROCHLORIDE	Small molecule	Histamine H1 receptor antagonist	Antagonist	HRH1	histamine receptor H1	4	Withdrawn	[illegible]
MONDO_0019353	EFO_0007141	allergic conjunctivitis	CHEMBL1201302	DEXAMETHASONE PHOSPHORIC ACID	Small molecule	Glucocorticoid receptor agonist		NR3C1	nuclear receptor subfamily 3 group C member	4		[illegible]
EFO_1001492	EFO_0007141	allergic conjunctivitis	CHEMBL516	CYPROHEPTADINE	Small molecule	Histamine H1 receptor antagonist	Antagonist	HRH1	histamine receptor H1	4		[illegible]
EFO_1001492	EFO_0007141	allergic conjunctivitis	CHEMBL1201759	BEPOTASTINE BESYLATE	Small molecule	Histamine H1 receptor antagonist	Antagonist	HRH1	histamine receptor H1	4	Completed	[illegible]
EFO_1001492	EFO_0007141	allergic conjunctivitis	CHEMBL1201353	DEXCHLORPHENIRAMINE	Small molecule	Histamine H1 receptor antagonist	Antagonist	HRH1	histamine receptor H1	4		[illegible]
EFO_1001492	EFO_0007141	allergic conjunctivitis	CHEMBL269732	TACROLIMUS	Small molecule	FK506-binding protein 1A inhibitor		FKBP1A	FKBP prolyl isomerase 1A	4	Completed	[illegible]
EFO_1001492	EFO_0007141	allergic conjunctivitis	CHEMBL1200750	PROMETHAZINE HYDROCHLORIDE	Small molecule	Histamine H1 receptor antagonist	Antagonist	HRH1	histamine receptor H1	4		[illegible]
EFO_0001365	EFO_0007141	allergic conjunctivitis	CHEMBL160	CYCLOSPORINE	Protein	Cyclophilin A modulator		PPIA	peptidylprolyl isomerase A	4	Completed	[illegible]
EFO_0001365	EFO_0007141	allergic conjunctivitis	CHEMBL1719	OLOPATADINE HYDROCHLORIDE	Small molecule	Histamine H1 receptor antagonist	Antagonist	HRH1	histamine receptor H1	4		[illegible]
EFO_1001492	EFO_0007141	allergic conjunctivitis	CHEMBL761	NAPHAZOLINE	Small molecule	Adrenergic receptor alpha agonist		ADRA2C	adrenoceptor alpha 2C	4	Unknown status	[illegible]
EFO_0001365	EFO_0007141	allergic conjunctivitis	CHEMBL1706	NAPHAZOLINE HYDROCHLORIDE	Small molecule	Adrenergic receptor alpha agonist		ADRA2C	adrenoceptor alpha 2C	4	Unknown status	[illegible]
EFO_1001492	EFO_0007141	allergic conjunctivitis	CHEMBL1706	NAPHAZOLINE HYDROCHLORIDE	Small molecule	Adrenergic receptor alpha agonist		ADRA2A	adrenoceptor alpha 2A	4	Unknown status	[illegible]
EFO_1001492	EFO_0007141	allergic conjunctivitis	CHEMBL1200809	AZELASTINE HYDROCHLORIDE	Small molecule	Histamine H1 receptor antagonist	Antagonist	HRH1	histamine receptor H1	4		[illegible]
EFO_0001365	EFO_0007141	allergic conjunctivitis	CHEMBL1106	EPINASTINE	Small molecule	Histamine H1 receptor antagonist	Antagonist	HRH1	histamine receptor H1	4	Withdrawn	[illegible]
EFO_0004683	EFO_0007141	allergic conjunctivitis	CHEMBL1201747	ALCAFTADINE	Small molecule	Histamine H1 receptor antagonist	Antagonist	HRH1	histamine receptor H1	4	Completed	[illegible]
EFO_1001492	EFO_0007141	allergic conjunctivitis	CHEMBL1719	OLOPATADINE HYDROCHLORIDE	Small molecule	Histamine H1 receptor antagonist	Antagonist	HRH1	histamine receptor H1	4	Completed	[illegible]
EFO_0001365	EFO_0007141	allergic conjunctivitis	CHEMBL1200491	EPINASTINE HYDROCHLORIDE	Small molecule	Histamine H1 receptor antagonist	Antagonist	HRH1	histamine receptor H1	4	Completed	[illegible]
EFO_1001492	EFO_0007141	allergic conjunctivitis	CHEMBL1200974	CARBINOXAMINE MALEATE	Small molecule	Histamine H1 receptor antagonist	Antagonist	HRH1	histamine receptor H1	4		[illegible]
EFO_0001365	EFO_0007141	allergic conjunctivitis	CHEMBL1706	NAPHAZOLINE HYDROCHLORIDE	Small molecule	Adrenergic receptor alpha agonist		ADRA2B	adrenoceptor alpha 2B	4	Unknown status	[illegible]
EFO_1001492	EFO_0007141	allergic conjunctivitis	CHEMBL761	NAPHAZOLINE	Small molecule	Adrenergic receptor alpha agonist		ADRA1B	adrenoceptor alpha 1B	4	Unknown status	[illegible]
EFO_1001492	EFO_0007141	allergic conjunctivitis	CHEMBL1172	DESLORATADINE	Small molecule	Histamine H1 receptor antagonist	Antagonist	HRH1	histamine receptor H1	4	Completed	[illegible]
EFO_0001365	EFO_0007141	allergic conjunctivitis	CHEMBL998	LORATADINE	Small molecule	Histamine H1 receptor antagonist	Antagonist	HRH1	histamine receptor H1	4	Completed	[illegible]
EFO_1001492	EFO_0007141	allergic conjunctivitis	CHEMBL1201758	BEPOTASTINE	Small molecule	Histamine H1 receptor antagonist	Antagonist	HRH1	histamine receptor H1	4	Completed	[illegible]
EFO_1001492	EFO_0007141	allergic conjunctivitis	CHEMBL1193	PHENIRAMINE	Small molecule	Histamine H1 receptor antagonist	Antagonist	HRH1	histamine receptor H1	4	Unknown status	[illegible]
EFO_1001492	EFO_0007141	allergic conjunctivitis	CHEMBL1473	FLUTICASONE PROPIONATE	Small molecule	Glucocorticoid receptor agonist		NR3C1	nuclear receptor subfamily 3 group C member	4	Completed	[illegible]
EFO_1001492	EFO_0007141	allergic conjunctivitis	CHEMBL1719	OLOPATADINE HYDROCHLORIDE	Small molecule	Histamine H1 receptor antagonist	Antagonist	HRH1	histamine receptor H1	4	Recruiting	[illegible]
EFO_0001365	EFO_0007141	allergic conjunctivitis	CHEMBL864	CARBINOXAMINE	Small molecule	Histamine H1 receptor antagonist	Antagonist	HRH1	histamine receptor H1	4		[illegible]
EFO_1001492	EFO_0007141	allergic conjunctivitis	CHEMBL1201191	LEVOCETIRIZINE	Small molecule	Histamine H1 receptor antagonist	Antagonist	HRH1	histamine receptor H1	4		[illegible]
EFO_0001365	EFO_0007141	allergic conjunctivitis	CHEMBL1706	NAPHAZOLINE HYDROCHLORIDE	Small molecule	Adrenergic receptor alpha agonist		ADRA1A	adrenoceptor alpha 1A	4	Unknown status	[illegible]
EFO_1001492	EFO_0007141	allergic conjunctivitis	CHEMBL269732	TACROLIMUS	Small molecule	FK506-binding protein 1A inhibitor		FKBP1A	FKBP prolyl isomerase 1A	4	Unknown status	[illegible]
EFO_1001492	EFO_0007141	allergic conjunctivitis	CHEMBL1189432	OLOPATADINE	Small molecule	Histamine H1 receptor antagonist	Antagonist	HRH1	histamine receptor H1	4	Withdrawn	[illegible]
EFO_1001492	EFO_0007141	allergic conjunctivitis	CHEMBL1152	PREDNISOLONE ACETATE	Small molecule	Glucocorticoid receptor agonist		NR3C1	nuclear receptor subfamily 3 group C member	4	Completed	[illegible]
EFO_0004683	EFO_0007141	allergic conjunctivitis	CHEMBL761	NAPHAZOLINE	Small molecule	Adrenergic receptor alpha agonist		ADRA1A	adrenoceptor alpha 1A	4	Unknown status	[illegible]
EFO_1001492	EFO_0007141	allergic conjunctivitis	CHEMBL534	KETOTIFEN	Small molecule	Histamine H1 receptor antagonist	Antagonist	HRH1	histamine receptor H1	4	Completed	[illegible]
EFO_1001492	EFO_0007141	allergic conjunctivitis	CHEMBL1201231	PREDNISOLONE PHOSPHORIC ACID	Small molecule	Glucocorticoid receptor agonist		NR3C1	nuclear receptor subfamily 3 group C member	4		[illegible]
EFO_1001492	EFO_0007141	allergic conjunctivitis	CHEMBL1706	NAPHAZOLINE HYDROCHLORIDE	Small molecule	Adrenergic receptor alpha agonist		ADRA1B	adrenoceptor alpha 1B	4	Unknown status	[illegible]
EFO_0001365	EFO_0007141	allergic conjunctivitis	CHEMBL1201014	PREDNISOLONE SODIUM PHOSPHATE	Small molecule	Glucocorticoid receptor agonist		NR3C1	nuclear receptor subfamily 3 group C member	4		[illegible]
EFO_0001365	EFO_0007141	allergic conjunctivitis	CHEMBL761	NAPHAZOLINE	Small molecule	Adrenergic receptor alpha agonist		ADRA1D	adrenoceptor alpha 1D	4	Unknown status	[illegible]
EFO_1001492	EFO_0007141	allergic conjunctivitis	CHEMBL1200386	PREDNICARBATE	Small molecule	Glucocorticoid receptor agonist		NR3C1	nuclear receptor subfamily 3 group C member	4		[illegible]
EFO_0001365	EFO_0007141	allergic conjunctivitis	CHEMBL1201231	PREDNISOLONE PHOSPHORIC ACID	Small molecule	Glucocorticoid receptor agonist		NR3C1	nuclear receptor subfamily 3 group C member	4	Completed	[illegible]
EFO_1001492	EFO_0007141	allergic conjunctivitis	CHEMBL1201758	BEPOTASTINE	Small molecule	Histamine H1 receptor antagonist	Antagonist	HRH1	histamine receptor H1	4	Unknown status	[illegible]
EFO_0004683	EFO_0007141	allergic conjunctivitis	CHEMBL1106	EPINASTINE	Small molecule	Histamine H1 receptor antagonist	Antagonist	HRH1	histamine receptor H1	4		[illegible]
EFO_0004683	EFO_0007141	allergic conjunctivitis	CHEMBL1201747	ALCAFTADINE	Small molecule	Histamine H1 receptor antagonist	Antagonist	HRH1	histamine receptor H1	4	Unknown status	[illegible]
EFO_1001492	EFO_0007141	allergic conjunctivitis	CHEMBL635	PREDNISONE	Small molecule	Glucocorticoid receptor agonist		NR3C1	nuclear receptor subfamily 3 group C member	4		[illegible]
EFO_1001492	EFO_0007141	allergic conjunctivitis	CHEMBL761	NAPHAZOLINE	Small molecule	Adrenergic receptor alpha agonist		ADRA2B	adrenoceptor alpha 2B	4	Unknown status	[illegible]
EFO_0004683	EFO_0007141	allergic conjunctivitis	CHEMBL1200953	HYDROCORTISONE PROBUTATE	Small molecule	Glucocorticoid receptor agonist		NR3C1	nuclear receptor subfamily 3 group C member	4		[illegible]
EFO_0004683	EFO_0007141	allergic conjunctivitis	CHEMBL1189432	OLOPATADINE	Small molecule	Histamine H1 receptor antagonist	Antagonist	HRH1	histamine receptor H1	4		[illegible]
EFO_0001365	EFO_0007141	allergic conjunctivitis	CHEMBL131	PREDNISOLONE	Small molecule	Glucocorticoid receptor agonist		NR3C1	nuclear receptor subfamily 3 group C member	4		[illegible]
EFO_1001492	EFO_0007141	allergic conjunctivitis	CHEMBL1653	PHENIRAMINE MALEATE	Small molecule	Histamine H1 receptor antagonist	Antagonist	HRH1	histamine receptor H1	4	Unknown status	[illegible]
EFO_0004683	EFO_0007141	allergic conjunctivitis	CHEMBL384467	DEXAMETHASONE	Small molecule	Glucocorticoid receptor agonist		NR3C1	nuclear receptor subfamily 3 group C member	4	Completed	[illegible]
EFO_1001492	EFO_0007141	allergic conjunctivitis	CHEMBL1152	PREDNISOLONE ACETATE	Small molecule	Glucocorticoid receptor agonist		NR3C1	nuclear receptor subfamily 3 group C member	4		[illegible]
EFO_1001492	EFO_0007141	allergic conjunctivitis	CHEMBL643	PROMETHAZINE	Small molecule	Histamine H1 receptor antagonist	Antagonist	HRH1	histamine receptor H1	4		[illegible]
EFO_1001492	EFO_0007141	allergic conjunctivitis	CHEMBL1189432	OLOPATADINE	Small molecule	Histamine H1 receptor antagonist	Antagonist	HRH1	histamine receptor H1	4	Recruiting	[illegible]
EFO_0001365	EFO_0007141	allergic conjunctivitis	CHEMBL1200491	EPINASTINE HYDROCHLORIDE	Small molecule	Histamine H1 receptor antagonist	Antagonist	HRH1	histamine receptor H1	4	Withdrawn	[illegible]
EFO_0001365	EFO_0007141	allergic conjunctivitis	CHEMBL1106	EPINASTINE	Small molecule	Histamine H1 receptor antagonist	Antagonist	HRH1	histamine receptor H1	4	Completed	[illegible]
EFO_0004683	EFO_0007141	allergic conjunctivitis	CHEMBL1201190	LEVOCETIRIZINE DIHYDROCHLORIDE	Small molecule	Histamine H1 receptor antagonist	Antagonist	HRH1	histamine receptor H1	4		[illegible]
EFO_1001492	EFO_0007141	allergic conjunctivitis	CHEMBL1676	FLUTICASONE FUROATE	Small molecule	Glucocorticoid receptor agonist		NR3C1	nuclear receptor subfamily 3 group C member	4	Completed	[illegible]
EFO_1001492	EFO_0007141	allergic conjunctivitis	CHEMBL2021430	DEXAMETHASONE SODIUM PHOSPHATE	Small molecule	Glucocorticoid receptor agonist		NR3C1	nuclear receptor subfamily 3 group C member	4		[illegible]
EFO_1001492	EFO_0007141	allergic conjunctivitis	CHEMBL1201759	BEPOTASTINE BESYLATE	Small molecule	Histamine H1 receptor antagonist	Antagonist	HRH1	histamine receptor H1	4		[illegible]
EFO_1001492	EFO_0007141	allergic conjunctivitis	CHEMBL1201747	ALCAFTADINE	Small molecule	Histamine H1 receptor antagonist	Antagonist	HRH1	histamine receptor H1	4		[illegible]
EFO_0004683	EFO_0007141	allergic conjunctivitis	CHEMBL1200927	DEXCHLORPHENIRAMINE MALEATE	Small molecule	Histamine H1 receptor antagonist	Antagonist	HRH1	histamine receptor H1	4		[illegible]
EFO_1001492	EFO_0007141	allergic conjunctivitis	CHEMBL761	NAPHAZOLINE	Small molecule	Adrenergic receptor alpha agonist		ADRA2A	adrenoceptor alpha 2A	4	Unknown status	[illegible]
EFO_1001492	EFO_0007141	allergic conjunctivitis	CHEMBL761	NAPHAZOLINE	Small molecule	Adrenergic receptor alpha agonist		ADRA2A	adrenoceptor alpha 2A	3	Completed	[illegible]
EFO_1001492	EFO_0007141	allergic conjunctivitis	CHEMBL1200491	EPINASTINE HYDROCHLORIDE	Small molecule	Histamine H1 receptor antagonist	Antagonist	HRH1	histamine receptor H1	3	Completed	[illegible]
EFO_1001492	EFO_0007141	allergic conjunctivitis	CHEMBL2062257	BRIMONIDINE TARTRATE	Small molecule	Adrenergic receptor alpha-2 agonist		ADRA2C	adrenoceptor alpha 2C	3	Recruiting	[illegible]
EFO_0001365	EFO_0007141	allergic conjunctivitis	CHEMBL1172	DESLORATADINE	Small molecule	Histamine H1 receptor antagonist	Antagonist	HRH1	histamine receptor H1	3	Completed	[illegible]
EFO_1001492	EFO_0007141	allergic conjunctivitis	CHEMBL1106	EPINASTINE	Small molecule	Histamine H1 receptor antagonist	Antagonist	HRH1	histamine receptor H1	3	Completed	[illegible]
EFO_1001492	EFO_0007141	allergic conjunctivitis	CHEMBL1077	BROMFENAC	Small molecule	Cyclooxygenase inhibitor		PTGS1	prostaglandin-endoperoxide synthase 1	3	Completed	[illegible]
EFO_1001492	EFO_0007141	allergic conjunctivitis	CHEMBL1719	OLOPATADINE HYDROCHLORIDE	Small molecule	Histamine H1 receptor antagonist	Antagonist	HRH1	histamine receptor H1	3	Completed	[illegible]
EFO_1001492	EFO_0007141	allergic conjunctivitis	CHEMBL1200865	LOTEPREDNOL ETABONATE	Small molecule	Glucocorticoid receptor agonist		NR3C1	nuclear receptor subfamily 3 group C member	3	Completed	[illegible]
EFO_1001492	EFO_0007141	allergic conjunctivitis	CHEMBL1189432	OLOPATADINE	Small molecule	Histamine H1 receptor antagonist	Antagonist	HRH1	histamine receptor H1	3	Completed	[illegible]
EFO_0001365	EFO_0007141	allergic conjunctivitis	CHEMBL2062257	BRIMONIDINE TARTRATE	Small molecule	Adrenergic receptor alpha-2 agonist		ADRA2B	adrenoceptor alpha 2B	3	Recruiting	[illegible]
EFO_1001492	EFO_0007141	allergic conjunctivitis	CHEMBL1189432	OLOPATADINE	Small molecule	Histamine H1 receptor antagonist	Antagonist	HRH1	histamine receptor H1	3	Withdrawn	[illegible]
EFO_1001492	EFO_0007141	allergic conjunctivitis	CHEMBL844	BRIMONIDINE	Small molecule	Adrenergic receptor alpha-2 agonist		ADRA2B	adrenoceptor alpha 2B	3	Recruiting	[illegible]
EFO_0001365	EFO_0007141	allergic conjunctivitis	CHEMBL1719	OLOPATADINE HYDROCHLORIDE	Small molecule	Histamine H1 receptor antagonist	Antagonist	HRH1	histamine receptor H1	3	Withdrawn	[illegible]
EFO_1001492	EFO_0007141	allergic conjunctivitis	CHEMBL384467	DEXAMETHASONE	Small molecule	Glucocorticoid receptor agonist		NR3C1	nuclear receptor subfamily 3 group C member	3	Completed	[illegible]
EFO_1001492	EFO_0007141	allergic conjunctivitis	CHEMBL1201759	BEPOTASTINE BESYLATE	Small molecule	Histamine H1 receptor antagonist	Antagonist	HRH1	histamine receptor H1	3	Completed	[illegible]
EFO_0004683	EFO_0007141	allergic conjunctivitis	CHEMBL269732	TACROLIMUS	Small molecule	FK506-binding protein 1A inhibitor		FKBP1A	FKBP prolyl isomerase 1A	3	Completed	[illegible]
EFO_1001492	EFO_0007141	allergic conjunctivitis	CHEMBL160	CYCLOSPORINE	Protein	Cyclophilin A modulator		PPIA	peptidylprolyl isomerase A	3	Completed	[illegible]
EFO_1001492	EFO_0007141	allergic conjunctivitis	CHEMBL1633	KETOTIFEN FUMARATE	Small molecule	Histamine H1 receptor antagonist	Antagonist	HRH1	histamine receptor H1	3	Completed	[illegible]
EFO_1001492	EFO_0007141	allergic conjunctivitis	CHEMBL2062257	BRIMONIDINE TARTRATE	Small molecule	Adrenergic receptor alpha-2 agonist		ADRA2A	adrenoceptor alpha 2A	3	Recruiting	[illegible]
EFO_1001492	EFO_0007141	allergic conjunctivitis	CHEMBL1077	BROMFENAC	Small molecule	Cyclooxygenase inhibitor		PTGS2	prostaglandin-endoperoxide synthase 2	3	Completed	[illegible]
EFO_1001492	EFO_0007141	allergic conjunctivitis	CHEMBL844	BRIMONIDINE	Small molecule	Adrenergic receptor alpha-2 agonist		ADRA2C	adrenoceptor alpha 2C	3	Recruiting	[illegible]
EFO_1001492	EFO_0007141	allergic conjunctivitis	CHEMBL761	NAPHAZOLINE	Small molecule	Adrenergic receptor alpha agonist		ADRA2B	adrenoceptor alpha 2B	3	Completed	[illegible]
EFO_0001365	EFO_0007141	allergic conjunctivitis	CHEMBL1742423	BILASTINE	Small molecule	Histamine H1 receptor inverse agonist	Antagonist	HRH1	histamine receptor H1	3	Completed	[illegible]
EFO_1001492	EFO_0007141	allergic conjunctivitis	CHEMBL761	NAPHAZOLINE	Small molecule	Adrenergic receptor alpha agonist		ADRA1B	adrenoceptor alpha 1B	3	Completed	[illegible]
EFO_0004683	EFO_0007141	allergic conjunctivitis	CHEMBL1000	CETIRIZINE	Small molecule	Histamine H1 receptor antagonist	Antagonist	HRH1	histamine receptor H1	3	Completed	[illegible]
EFO_0001365	EFO_0007141	allergic conjunctivitis	CHEMBL761	NAPHAZOLINE	Small molecule	Adrenergic receptor alpha agonist		ADRA1A	adrenoceptor alpha 1A	3	Completed	[illegible]
EFO_0004683	EFO_0007141	allergic conjunctivitis	CHEMBL844	BRIMONIDINE	Small molecule	Adrenergic receptor alpha-2 agonist		ADRA2A	adrenoceptor alpha 2A	3	Recruiting	[illegible]
EFO_0001365	EFO_0007141	allergic conjunctivitis	CHEMBL761	NAPHAZOLINE	Small molecule	Adrenergic receptor alpha agonist		ADRA1D	adrenoceptor alpha 1D	3	Completed	[illegible]
EFO_1001492	EFO_0007141	allergic conjunctivitis	CHEMBL534	KETOTIFEN	Small molecule	Histamine H1 receptor antagonist	Antagonist	HRH1	histamine receptor H1	3	Recruiting	[illegible]
EFO_0001365	EFO_0007141	allergic conjunctivitis	CHEMBL761	NAPHAZOLINE	Small molecule	Adrenergic receptor alpha agonist		ADRA2C	adrenoceptor alpha 2C	3	Completed	[illegible]
EFO_0004683	EFO_0007141	allergic conjunctivitis	CHEMBL534	KETOTIFEN	Small molecule	Histamine H1 receptor antagonist	Antagonist	HRH1	histamine receptor H1	3	Completed	[illegible]
EFO_0001365	EFO_0007141	allergic conjunctivitis	CHEMBL1633	KETOTIFEN FUMARATE	Small molecule	Histamine H1 receptor antagonist	Antagonist	HRH1	histamine receptor H1	3	Recruiting	[illegible]
EFO_1001492	EFO_0007141	allergic conjunctivitis	CHEMBL1201758	BEPOTASTINE	Small molecule	Histamine H1 receptor antagonist	Antagonist	HRH1	histamine receptor H1	3	Completed	[illegible]
EFO_1001492	EFO_0007141	allergic conjunctivitis	CHEMBL1201747	ALCAFTADINE	Small molecule	Histamine H1 receptor antagonist	Antagonist	HRH1	histamine receptor H1	3	Completed	[illegible]
EFO_1001492	EFO_0007141	allergic conjunctivitis	CHEMBL1200791	OXYMETAZOLINE HYDROCHLORIDE	Small molecule	Adrenergic receptor alpha agonist		ADRA2C	adrenoceptor alpha 2C	2	Completed	[illegible]
EFO_0004683	EFO_0007141	allergic conjunctivitis	CHEMBL1152	PREDNISOLONE ACETATE	Small molecule	Glucocorticoid receptor agonist		NR3C1	nuclear receptor subfamily 3 group C member	2	Completed	[illegible]
EFO_1001492	EFO_0007141	allergic conjunctivitis	CHEMBL2103876	MAPRACORAT	Small molecule	Glucocorticoid receptor agonist		NR3C1	nuclear receptor subfamily 3 group C member	2	Completed	[illegible]
EFO_1001492	EFO_0007141	allergic conjunctivitis	CHEMBL2062257	BRIMONIDINE TARTRATE	Small molecule	Adrenergic receptor alpha-2 agonist		ADRA2A	adrenoceptor alpha 2A	2	Completed	[illegible]
EFO_1001492	EFO_0007141	allergic conjunctivitis	CHEMBL1189432	OLOPATADINE	Small molecule	Histamine H1 receptor antagonist	Antagonist	HRH1	histamine receptor H1	2	Completed	[illegible]
EFO_0004683	EFO_0007141	allergic conjunctivitis	CHEMBL534	KETOTIFEN	Small molecule	Histamine H1 receptor antagonist	Antagonist	HRH1	histamine receptor H1	2	Withdrawn	[illegible]
EFO_0004683	EFO_0007141	allergic conjunctivitis	CHEMBL389621	HYDROCORTISONE	Small molecule	Glucocorticoid receptor agonist		NR3C1	nuclear receptor subfamily 3 group C member	2	Terminated	[illegible]
EFO_0001365	EFO_0007141	allergic conjunctivitis	CHEMBL269732	TACROLIMUS	Small molecule	FK506-binding protein 1A inhibitor		FKBP1A	FKBP prolyl isomerase 1A	2	Unknown status	[illegible]
EFO_1001492	EFO_0007141	allergic conjunctivitis	CHEMBL1201438	ALDESLEUKIN	Protein	Interleukin-2 receptor agonist		IL2RB	interleukin 2 receptor subunit beta	2	Completed	[illegible]
EFO_0001365	EFO_0007141	allergic conjunctivitis	CHEMBL384467	DEXAMETHASONE	Small molecule	Glucocorticoid receptor agonist		NR3C1	nuclear receptor subfamily 3 group C member	2	Withdrawn	[illegible]
EFO_0001365	EFO_0007141	allergic conjunctivitis	CHEMBL1201759	BEPOTASTINE BESYLATE	Small molecule	Histamine H1 receptor antagonist	Antagonist	HRH1	histamine receptor H1	2	Completed	[illegible]
EFO_1001492	EFO_0007141	allergic conjunctivitis	CHEMBL269732	TACROLIMUS	Small molecule	FK506-binding protein 1A inhibitor		FKBP1A	FKBP prolyl isomerase 1A	2	Completed	[illegible]
EFO_0001365	EFO_0007141	allergic conjunctivitis	CHEMBL762	OXYMETAZOLINE	Small molecule	Adrenergic receptor alpha agonist		ADRA1D	adrenoceptor alpha 1D	2	Completed	[illegible]
EFO_0004683	EFO_0007141	allergic conjunctivitis	CHEMBL1200791	OXYMETAZOLINE HYDROCHLORIDE	Small molecule	Adrenergic receptor alpha agonist		ADRA1A	adrenoceptor alpha 1A	2	Completed	[illegible]
EFO_1001492	EFO_0007141	allergic conjunctivitis	CHEMBL1719	OLOPATADINE HYDROCHLORIDE	Small molecule	Histamine H1 receptor antagonist	Antagonist	HRH1	histamine receptor H1	2	Completed	[illegible]
EFO_1001492	EFO_0007141	allergic conjunctivitis	CHEMBL2048028	LIFITEGRAST	Small molecule	Integrin alpha-L/beta-2 (LFA-1) antagonist		ITGB2	integrin subunit beta 2	2	Completed	[illegible]
EFO_1001492	EFO_0007141	allergic conjunctivitis	CHEMBL762	OXYMETAZOLINE	Small molecule	Adrenergic receptor alpha agonist		ADRA1B	adrenoceptor alpha 1B	2	Completed	[illegible]
EFO_1001492	EFO_0007141	allergic conjunctivitis	CHEMBL1742423	BILASTINE	Small molecule	Histamine H1 receptor inverse agonist	Antagonist	HRH1	histamine receptor H1	2	Completed	[illegible]
EFO_1001492	EFO_0007141	allergic conjunctivitis	CHEMBL1000	CETIRIZINE	Small molecule	Histamine H1 receptor antagonist	Antagonist	HRH1	histamine receptor H1	2	Completed	[illegible]
EFO_0004683	EFO_0007141	allergic conjunctivitis	CHEMBL762	OXYMETAZOLINE	Small molecule	Adrenergic receptor alpha agonist		ADRA2B	adrenoceptor alpha 2B	2	Completed	[illegible]
EFO_0004683	EFO_0007141	allergic conjunctivitis	CHEMBL635	PREDNISONE	Small molecule	Glucocorticoid receptor agonist		NR3C1	nuclear receptor subfamily 3 group C member	2	Completed	[illegible]
EFO_0004683	EFO_0007141	allergic conjunctivitis	CHEMBL2062257	BRIMONIDINE TARTRATE	Small molecule	Adrenergic receptor alpha-2 agonist		ADRA2B	adrenoceptor alpha 2B	2	Completed	[illegible]
EFO_0001365	EFO_0007141	allergic conjunctivitis	CHEMBL1200865	LOTEPREDNOL ETABONATE	Small molecule	Glucocorticoid receptor agonist		NR3C1	nuclear receptor subfamily 3 group C member	2	Completed	[illegible]
EFO_0004683	EFO_0007141	allergic conjunctivitis	CHEMBL1200791	OXYMETAZOLINE HYDROCHLORIDE	Small molecule	Adrenergic receptor alpha agonist		ADRA2A	adrenoceptor alpha 2A	2	Completed	[illegible]
EFO_0001365	EFO_0007141	allergic conjunctivitis	CHEMBL384467	DEXAMETHASONE	Small molecule	Glucocorticoid receptor agonist		NR3C1	nuclear receptor subfamily 3 group C member	2	Completed	[illegible]
EFO_0004683	EFO_0007141	allergic conjunctivitis	CHEMBL2108675	DUPILUMAB	Antibody	Interleukin-4 receptor subunit alpha antagonist		IL4R	interleukin 4 receptor	2	Recruiting	[illegible]
EFO_0004683	EFO_0007141	allergic conjunctivitis	CHEMBL1201758	BEPOTASTINE	Small molecule	Histamine H1 receptor antagonist	Antagonist	HRH1	histamine receptor H1	2	Completed	[illegible]
EFO_0004683	EFO_0007141	allergic conjunctivitis	CHEMBL1200791	OXYMETAZOLINE HYDROCHLORIDE	Small molecule	Adrenergic receptor alpha agonist		ADRA1D	adrenoceptor alpha 1D	2	Completed	[illegible]
EFO_0004683	EFO_0007141	allergic conjunctivitis	CHEMBL844	BRIMONIDINE	Small molecule	Adrenergic receptor alpha-2 agonist		ADRA2B	adrenoceptor alpha 2B	2	Completed	[illegible]
EFO_0004683	EFO_0007141	allergic conjunctivitis	CHEMBL1201438	ALDESLEUKIN	Protein	Interleukin-2 receptor agonist		IL2RA	interleukin 2 receptor subunit alpha	2	Completed	[illegible]
EFO_0004683	EFO_0007141	allergic conjunctivitis	CHEMBL1189432	OLOPATADINE	Small molecule	Histamine H1 receptor antagonist	Antagonist	HRH1	histamine receptor H1	2	Terminated	[illegible]
EFO_0004683	EFO_0007141	allergic conjunctivitis	CHEMBL534	KETOTIFEN	Small molecule	Histamine H1 receptor antagonist	Antagonist	HRH1	histamine receptor H1	2	Completed	[illegible]
EFO_0001365	EFO_0007141	allergic conjunctivitis	CHEMBL762	OXYMETAZOLINE	Small molecule	Adrenergic receptor alpha agonist		ADRA1A	adrenoceptor alpha 1A	2	Completed	[illegible]
EFO_0004683	EFO_0007141	allergic conjunctivitis	CHEMBL160	CYCLOSPORINE	Protein	Cyclophilin A modulator		PPIA	peptidylprolyl isomerase A	2	Completed	[illegible]
EFO_0004683	EFO_0007141	allergic conjunctivitis	CHEMBL844	BRIMONIDINE	Small molecule	Adrenergic receptor alpha-2 agonist		ADRA2A	adrenoceptor alpha 2A	2	Completed	[illegible]
EFO_0001365	EFO_0007141	allergic conjunctivitis	CHEMBL2062257	BRIMONIDINE TARTRATE	Small molecule	Adrenergic receptor alpha-2 agonist		ADRA2C	adrenoceptor alpha 2C	2	Completed	[illegible]
EFO_0004683	EFO_0007141	allergic conjunctivitis	CHEMBL2048028	LIFITEGRAST	Small molecule	Integrin alpha-L/beta-2 (LFA-1) antagonist		ITGAL	integrin subunit alpha L	2	Completed	[illegible]
EFO_0004683	EFO_0007141	allergic conjunctivitis	CHEMBL762	OXYMETAZOLINE	Small molecule	Adrenergic receptor alpha agonist		ADRA2A	adrenoceptor alpha 2A	2	Completed	[illegible]
EFO_0001365	EFO_0007141	allergic conjunctivitis	CHEMBL1042	CHOLECALCIFEROL	Small molecule	Vitamin D receptor agonist		VDR	vitamin D receptor	2	Completed	[illegible]
EFO_0001365	EFO_0007141	allergic conjunctivitis	CHEMBL844	BRIMONIDINE	Small molecule	Adrenergic receptor alpha-2 agonist		ADRA2C	adrenoceptor alpha 2C	2	Completed	[illegible]
EFO_0004683	EFO_0007141	allergic conjunctivitis	CHEMBL1200791	OXYMETAZOLINE HYDROCHLORIDE	Small molecule	Adrenergic receptor alpha agonist		ADRA1B	adrenoceptor alpha 1B	2	Completed	[illegible]
EFO_0004683	EFO_0007141	allergic conjunctivitis	CHEMBL1200791	OXYMETAZOLINE HYDROCHLORIDE	Small molecule	Adrenergic receptor alpha agonist		ADRA2B	adrenoceptor alpha 2B	2	Completed	[illegible]
EFO_0001365	EFO_0007141	allergic conjunctivitis	CHEMBL762	OXYMETAZOLINE	Small molecule	Adrenergic receptor alpha agonist		ADRA2C	adrenoceptor alpha 2C	2	Completed	[illegible]
EFO_0001365	EFO_0007141	allergic conjunctivitis	CHEMBL534	KETOTIFEN	Small molecule	Histamine H1 receptor antagonist	Antagonist	HRH1	histamine receptor H1	1	Completed	[illegible]
	EFO_0007141	allergic conjunctivitis	CHEMBL1200865	LOTEPREDNOL ETABONATE	Small molecule	Glucocorticoid receptor agonist		NR3C1	nuclear receptor subfamily 3 group C member	1	Completed	[illegible]
	EFO_0007141	allergic conjunctivitis	CHEMBL160	CYCLOSPORINE	Protein	Cyclophilin A modulator		PPIA	peptidylprolyl isomerase A	1	Completed	[illegible]
	EFO_0007141	allergic conjunctivitis	CHEMBL1189432	OLOPATADINE	Small molecule	Histamine H1 receptor antagonist	Antagonist	HRH1	histamine receptor H1	1	Completed	[illegible]
	EFO_0007141	allergic conjunctivitis	CHEMBL4297878	LIRENTELIMAB	Antibody	Sialic acid-binding Ig-like lectin 8 inhibitor		SIGLEC8	sialic acid binding Ig like lectin 8	1	Completed	[illegible]
	EFO_0007141	allergic conjunctivitis	CHEMBL1633	KETOTIFEN FUMARATE	Small molecule	Histamine H1 receptor antagonist	Antagonist	HRH1	histamine receptor H1	1	Completed	[illegible]
